# Prevalence of Genetic Variants Associated with Atrial Fibrillation Risk in the Asymptomatic Young Adult Population

**DOI:** 10.3390/medicina61050900

**Published:** 2025-05-15

**Authors:** Manoranjani Murugan, Sambandam Ravikumar, Irisappan Ganesh, Yogesh Vetriselvan, Arunagiri Priyadharshini, Vishnu Bhat Ballambattu

**Affiliations:** 1 Department of Medical Biotechnology, Aarupadai Veedu Medical College and Hospital, Vinayaka Mission’s Research Foundation (Deemed to be University), Kirumampakkam, Puducherry 607403, India; manoranjanimurugan@avmc.edu.in (M.M.); ravikumar.sambandam@avmc.edu.in (S.R.); ganesh.irisappan@avmc.edu.in (I.G.); yogeshvetriselvan@avmc.edu.in (Y.V.); 2 Department of Biochemistry, Aarupadai Veedu Medical College and Hospital, Vinayaka Mission’s Research Foundation (Deemed to be University), Kirumampakkam, Puducherry 607403, India; priyaarunagiri002@gmail.com; 3 Advisor—Medical Research and Publications, Aarupadai Veedu Medical College and Hospital, Vinayaka Mission’s Research Foundation (Deemed to be University), Kirumampakkam, Puducherry 607403, India

**Keywords:** atrial fibrillation, genetic variants, transcription factors, young adults, SNP

## Abstract

*Background and Objectives*: Atrial fibrillation (AF) is the most common cardiac arrhythmia globally, leading to a high risk of stroke and heart failure. Genetic factors are known to play an essential role in AF risk. However, studies on genetic predisposition in asymptomatic young populations remain limited. This study aimed to investigate the prevalence of genetic variants in the *PITX2* (rs2200733, rs10033464, and rs13143308), *TBX5* (rs883079), *PRRX1* (rs3903239), *ZFHX3* (rs2106261), and *HAND2* (rs7698692) polymorphisms and to assess their correlation with susceptibility to AF in a young adult population in India. *Materials and Methods*: This cross-sectional study included 250 subjects aged 18–29. Detailed lifestyle and family histories were collected for each participant. Genetic variation was determined using a specific TaqMan SNP genotyping assay. Hardy–Weinberg equilibrium (HWE) analysis and chi-square tests were employed to assess genotype frequencies, and statistical associations with lifestyle factors (body mass index, alcohol consumption, and smoking) were evaluated using *t*-tests and descriptive statistics. *Results*: Minor allele frequencies were varied across the study population, with notable frequencies in rs2200733 T (16%), rs10033464 T (27%), rs13143308 T (32%), rs883079 T (46%), rs3903239 G (25%), rs2106261 T (26%), and rs7698692 G (14%). HWE analysis confirmed that all SNPs were in equilibrium (*p* > 0.05). Approximately 15% of individuals carried six or more risk alleles, indicating a significant genetic predisposition to AF despite the absence of clinical symptoms. *Conclusions*: This study provides new insights into the genetic predisposition to AF among young adults in India. The high prevalence of risk alleles in asymptomatic young adults highlights the necessity of early genetic screening for AF risk and the role of genetic counseling in preventing cardiac complications.

## 1. Introduction

Atrial fibrillation (AF) is the most predominant cardiac arrhythmia, affecting more than 33 million people worldwide, which leads to significant complications like stroke and heart failure [1,2,3]. AF is characterized by an irregular heartbeat, changes in heart tissue, or interruptions in the heart’s electrical signal [4,5]. These irregularities lead to irregular contractions of the heart’s upper chamber, resulting in the pooling of blood and the formation of clots. These complications significantly increase the risk of stroke, which is the leading cause of disability and mortality across the world [3,4,6]. Although AF is uncommon in individuals under 40, it causes unique risks even in younger populations. Recent studies conducted at tertiary care hospitals in eastern India have indicated that younger individuals with AF also have other related problems like systemic hypertension, left ventricular systolic dysfunction, and metabolic and structural heart disease [7]. These associated conditions can complicate the clinical management of AF and highlight the need for a deeper understanding of the conditions in this population. In India, AF constitutes approximately 0.5%, but studies on its prevalence among younger adults are limited [8].

Recent studies suggest that genetic factors significantly influence the hereditary risk of developing AF. Specific single-nucleotide polymorphisms (SNPs) increase the risk of susceptibility to AF [9,10,11]. Genome-wide association studies (GWASs) have identified several SNPs located near the essential genes *PITX2*, *TBX5*, *PRRX1*, *ZFHX3*, *and HAND2*, which are involved in cardiac development and function [12,13]. The genetic variants, such as rs2200733, rs10033464, rs13143308, rs883079, rs3903239, rs2106261, and rs7698692, are located near essential genes and are significantly associated with an elevated risk of developing AF [11,14,15,16,17,18]. These variants influence atrial electrical activity and play an essential role in the development of the heart, contributing to AF susceptibility. The genetic variants rs2200733, rs10033464, and rs13143308 are located near the *PITX2* gene, which regulates atrial electrical conduction [9,19]; the genetic variant of rs883079 located in 3′ UTR of the *TBX5* gene has been associated with atrial activation patterns; and *TBX5* directly activates the *PITX2* gene [20]. The genetic variants in *PRRX1* (rs3903239), *ZFHX3* (rs2106261), and *HAND2* (rs7698692) modulate the electrical activity and structural development of the heart, influencing AF susceptibility [21]. Although the genetic factors’ association with AF has been identified, most AF-based genetic research has focused only on older and symptomatic populations. This opens up a knowledge gap in understanding the genetic predisposition to AF among younger and asymptomatic individuals. Studying genetic variants in the young adult population diagnoses early AF risk, enabling targeted prevention, personalized treatments, and a better understanding of gene–lifestyle interactions to mitigate AF-related problems.

To develop preventive and customized medical strategies for AF, genetic testing is highly effective in identifying monogenic defects. A genetic predisposition is likely to play an important role in cases of early-onset AF where specific cardiovascular risk factors are absent. Studying genetic variation among younger adults is necessary for the early identification of genetic risks, allowing for more timely actions to modify lifestyle or increase monitoring to mitigate disease progression. Additionally, focusing on younger populations helps to assess the influence of genetics on age-related comorbidities, thereby enhancing the precision of investigating the genetic causative factors. However, genetic research remains a powerful tool for diagnosing, stratifying risks, and managing AF, despite challenges posed by variants of unknown significance and the low diversity of participants. This research fills a significant gap in the genetics of AF among young adults by enhancing the biomarker resources customized for specific age demographics. Focusing on this young group uncovers non-standard risk factors, enables early AF detection, predicts progression, and informs management.

Herein, we aim to investigate the distribution of AF-related SNPs in the young adult population in India, who are also asymptomatic for AF. Through this analysis, we hope to identify individuals with high-risk genetic profiles who may benefit from early intervention to prevent AF complications. Additionally, this study aims to determine the frequency and distribution of SNPs associated with AF, focusing on their impact on heart health in young adults. Although most AF research on genetic variants focuses on older populations where AF is more prevalent, our study offers novel insights into variants that may influence AF risk at a younger age, particularly in individuals without risk factors for AF, such as hypertension or structural heart disease. Thus, in this study, we assessed the prevalence of both high-risk and protective alleles, particularly in the Indian community, which contributes to enhancing our understanding of the genetic predisposition to AF among younger individuals.

## 2. Materials and Methods

### 2.1. Study Subjects

This observational study was conducted among 250 young adults recruited from the students and staff of Aarupadai Veedu Medical College & Hospital, Puducherry. The inclusion criterion for this study’s participants was young adults aged between 18 and 29 years. We obtained written informed consent from all participants and confirmed their willingness to complete a comprehensive questionnaire that addressed their medical history, family history, and lifestyle habits. This study was approved by our institution’s Human Ethics Committee (IEC No: AV/IEC/2020/022). Height, weight, and hip circumference measurements were taken to calculate the body mass index (BMI). For clarification, a BMI ranging from 18.5 to 24.9 was categorized as within the normal weight range, a BMI from 25.0 to 29.9 was classified as overweight, and a BMI of 30.0 or greater as obesity. Participants were excluded from the study if they had a documented history of cardiovascular disease, metabolic disorders, or chronic illnesses. Individuals receiving medications that impact metabolic parameters, including steroids, antidiabetic agents, or antihypertensive medications, were not included. Furthermore, pregnant or lactating women, individuals who had undergone an acute illness or hospitalization within the prior three months, as well as those unwilling to provide a blood sample or complete consent forms, were excluded. Two ml of blood sample was obtained from each study participant, and the samples were either refrigerated for storage or processed immediately for DNA extraction.

### 2.2. DNA Isolation

The human genomic DNA was isolated from 200 μL of blood sample using the QIAamp Blood Mini Kit (Qiagen, Hilden, Germany) according to the manufacturer’s instructions. The isolated DNA samples were stored at −20 °C till further analysis was conducted. The quality of the isolated DNA was checked using agarose gel electrophoresis. The DNA concentration was quantified using a Nanodrop (BioPhotometer D30, Eppendorf AG, Hamburg, Germany).

### 2.3. Genotyping via Real-Time PCR

The *PITX2* (rs2200733, rs10033464, and rs13143308), *TBX5* (rs883079), *PRRX1* (rs3903239), *ZFHX3* (rs2106261), and *HAND2* (rs7698692) allele frequencies were determined using a TaqMan SNP genotyping assay via quantitative real-time PCR. The PCR reaction mixture consisted of 5 μL of TaqMan genotyping PCR master mix, 0.5 μL of TaqMan genotyping assay mix, and 20 ng of purified genomic DNA in a total volume of 10 μL of DNase-free water. The real-time PCR conditions for amplification were 10 min at 95 °C, followed by 40 cycles of denaturation for 15 s at 95 °C and annealing/extension for 1 min at 60 °C. The fluorescent signal intensities were recorded, and genotyping was analyzed using Quant Studio 5 detector systems (Applied Biosystems, Thermo Fisher Scientific Inc., Waltham, MA, USA) software. The SNPs in various genes, their SNP IDs, allele changes, SNP types, and the corresponding context sequences, are presented in Table 1.

### 2.4. Hardy–Weinberg Equilibrium Analysis

Hardy–Weinberg equilibrium (HWE) was used to calculate the SNP genotype frequencies, which were then compared with the expected frequencies using the chi-square (χ^2^) test. A *p*-value of less than 0.05 was considered statistically significant, indicating a deviation from the Hardy–Weinberg equilibrium (HWE).

### 2.5. Variant Allele Frequency Analysis

The allele frequencies of the rs2200733, rs10033464, rs13143308, rs883079, rs3903239, rs2106261, and rs7698692 genetic variants were calculated and compared with those of publicly available databases, including the 1000 Genomes Project, gnomAD, and IndiGenome. Frequencies in the study population were derived via allele counting and expressed as a proportion of the total number of alleles at each locus.

### 2.6. Risk Allele Categorization

We evaluated each participant for the number of risk alleles across the selected seven SNPs—rs2200733, rs10033464, rs13143308, rs883079, rs3903239, rs2106261, and rs7698692—which are associated with a greater susceptibility to AF. Participants were categorized into three groups based on their total count of risk alleles: low risk (0–3 risk alleles), medium risk (4–5 risk alleles), and high risk (6–8 risk alleles). With this classification, steps can be taken to personalize preventive strategies that require different intervention levels depending on the individual’s risk.

### 2.7. Association Between Risk Allele Categories and Lifestyle Factors

The study participants’ height and weight were measured, and their BMI was calculated using standard methodologies and defined as normal (BMI < 25), overweight (BMI 25–29.9), or obese (BMI ≥ 30). Data on the habits of alcohol consumption and smoking were also collected. The risk allele category group was compared based on obesity, alcohol consumption, and smoking status to determine the contribution of lifestyle factors to disease progression.

### 2.8. Statistical Analysis

The obtained data were presented as mean ± standard deviation (SD) for continuous variables, and categorical variables were represented as numbers and percentages. The Student’s *t*-test was used to compare the obtained quantitative variables. The allelic frequency, genotypic frequency, and descriptive statistics tables were generated from the received data using Microsoft Office 2019. The Hardy–Weinberg equilibrium was analyzed using a chi-square test (χ^2^) with SPSS 28.0 (IBM, Chicago, IL, USA). A *p*-value of less than 0.05 was considered statistically significant.

## 3. Results

### 3.1. Demographic Characteristics of the Young Adult Population

A total of 250 young adults, with a mean age of 25.81 years (SD ± 6.19), participated in this study. Among the participants, 31% were female and 69% were male. The average body mass index (BMI) was 24.9 kg/m^2^ (SD ± 4.2). Among them, 38% of the population was classified as overweight, 12% as obese, and 50% as normal. Concerning lifestyle habits, 36% did not consume alcohol. Among them, 21% were non-smokers. Considering family medical history, 17% of the study participants had a heritable predisposition to obesity, 15% to cardiovascular disease, and 23% to diabetes mellitus. The demographic profile of the study population is shown in Table 2.

### 3.2. Distribution of Genotypic Frequency in the Young Adult Population

The *PITX2* (rs2200733, rs10033464, and rs13143308) homozygous mutant genotype’s (TT, TT, and TT) frequencies were 3%, 13%, and 10%, and its minor allele (T, T, and T) frequencies were 16%, 27%, and 32%. The *TBX5* (rs883079) homozygous mutant genotype’s (TT) frequency was 21%, and its minor allele (T) frequency was 46%. The *PRRX1* (rs3903239) homozygous mutant genotype’s (GG) frequency was 7%, and the frequency of its minor allele (T) was 25%. The frequency of the *ZFHX3* (rs2106261) homozygous mutant genotype (TT) was 7%, and the frequency of its minor allele (T) was 26%. The *HAND2* (rs2106261) homozygous mutant genotype’s (GG) frequency was 1%, and its minor allele’s (G) frequency was 14%. The genotype and allele distributions of SNPs associated with AF are presented in Table 3. Detailed information on the genotyping results with the HWE values of the study group is shown in the Supplementary Table S1. The comparison of the variant allele frequency in the South Asian population with that in the 1000 Genomes, Genome AD, and IndiGenome databases is shown in Table 4.

### 3.3. Distribution of Risk Alleles in the Young Adult Population

To study the interaction among SNPs—including rs2200733, rs10033464, rs13143308, rs3903239, rs2106261, rs883079, and rs7698692—the prevalence of risk alleles for AF was examined in the young adult population of India. The distribution of risk alleles in the study population is shown in Table 5. The results show that the proportion of risk alleles in young adults (n = 250) varied among individuals. Only 3% of participants possessed no risk alleles, while 9% carried one risk allele and 15% carried two. Furthermore, 19% of the population had three risk alleles, and the largest group, encompassing 20% of participants, had four. Although 19% of the study group carried five risk alleles, higher risk alleles were observed in 7% with six risk alleles, 4% with seven risk alleles, and 3% with eight risk alleles.

### 3.4. Risk Allele Association with Obesity and Alcohol

**Figure 1:**
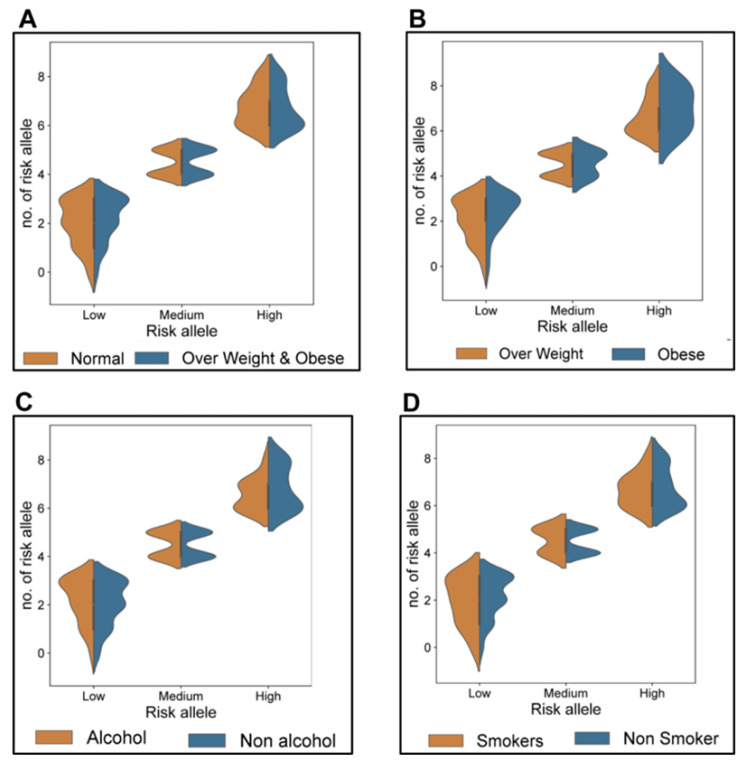
Comparison of risk allele distribution between BMI, alcohol consumption, and smoking individuals: The violin plot compares the distribution of risk alleles between two groups, such as (**A**) normal and overweight individuals, (**B**) overweight and obese individuals, (**C**) alcohol and non-alcohol individuals, and (**D**) smokers and non-smokers. Here, the x-axis indicates the different categories of risk alleles, such as low, medium, and high. The y-axis denotes the number of risk alleles, ranging from 0 to 8. The density of individuals possessing the specific number of risk alleles within each group is represented by the width of the violin. A wider range denotes a greater number of individuals possessing risk alleles.

**Figure 2:**
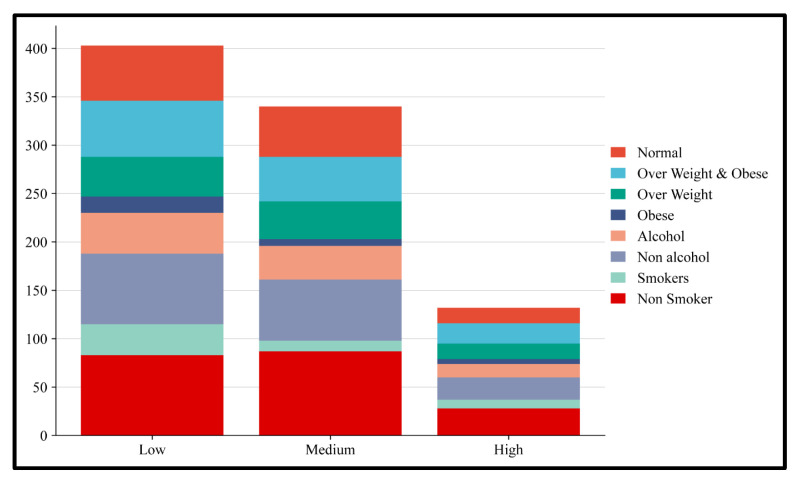
Distribution of BMI categories, alcohol consumption, and smoking status in the young adult population. A stacked bar chart shows the distribution of health-related behavioral and physiological traits: BMI (Normal, Overweight, and Obese), alcohol consumption (Alcohol and Non-alcohol), and smoking status (Smokers and Non-Smokers) among Low, Medium, and High-risk groups. Each color block represents a category. The chart reveals lifestyle and health indicator differences between the three groups, with the Low group showing higher counts in many risk-related categories.

The risk alleles were further categorized into high, medium, and low-risk alleles. Among them, alleles 6, 7, and 8 were classified as high risk, alleles 4 and 5 were considered medium risk, and alleles 0, 1, 2, and 3 were considered low risk. These three groups of risk alleles were then associated with BMI, alcohol consumption, and smoking habits to assess genetic and lifestyle factors. From the results obtained, we found that 8% of individuals combining both the overweight and obese groups had a high-risk allele, whereas only 5% of the obese group had a high-risk allele. We also found that 6% of the alcohol-consuming group and 9% of the smoking group carried the high-risk allele (Figure 1 and Figure 2).

## 4. Discussion

Atrial fibrillation (AF) is predominantly considered an abnormal heart condition that affects mainly older people, with the risks considerably increasing with age [22]. Common conditions that increase the risk of AF include chronic kidney disease [23], diabetes [24], high blood pressure [25], overweight and obesity [26], hyperthyroidism, COPD, and other lung problems [27]. Furthermore, mutations in the genes related to the ion channels or structural proteins of the heart are associated with early-onset AF [9,28,29]. In addition to the genetic causes mentioned, conditions such as congenital heart disease [30,31], cardiomyopathies [32], and inherited syndromes (such as Brugada syndrome or Long QT syndrome) are also associated with an increased risk of AF in the young population [33,34,35]. In addition, lifestyle habits like high-intensity exercise, excessive alcohol or use of stimulants, and underlying disorders like obesity or sleep apnea cause increased susceptibility to AF in younger individuals [36]. A further literature review also suggests that younger individuals are more susceptible to AF, which is caused by genetic factors and other associated risk determinants [37,38]. This study investigated the prevalence of genetic variants associated with AF in the young adult population in India. Here, we focused on common single-nucleotide polymorphisms (SNPs) in transcription factors, including *PITX2* (rs2200733, rs10033464, and rs13143308), *TBX5* (rs883079), *PRRX1* (rs3903239), *ZFHX3* (rs2106261), and *HAND2* (rs7698692). GWASs have shown that these genetic variants are linked to susceptibility to AF [39]. The findings of this study provide the prevalence and distribution of risk alleles within the young population, offering valuable insights into AF risk, particularly in the Indian community.

Genetic variants, such as rs2200733, rs10033464, and rs13143308, located on chromosome 4q25 near the *PITX2* gene, are highly associated with AF. Previous studies have consistently highlighted the *PITX2* gene as a key locus for AF development, particularly in European populations [11,40]. Our analysis identified the prevalence of *PITX2* variants in the young adult population in India. The *PITX2* gene is essential for cardiac development and function, especially in regulating left–right asymmetry and atrial electrical activity. The proximity of the variants to the *PITX2* gene suggests that they influence atrial electrical remodeling and conduction, predisposing individuals to AF [9,41]. In this study, the high prevalence of the rs883079 variant (Table 2), located on chromosome 12 at the 3′ UTR of the *TBX5* gene, influences the AF risk, consistent with previous findings in other ethnic groups [17]. Additionally, this variant is associated with a prolonged PR interval, which serves as an index of electrical activation from the atrial chambers [17]. The prevalence of the rs3903239 variant (Table 2), located on chromosome 1q24, 46 kb upstream of the PRRX1 gene, has been associated with a lower incidence of AF. The presence of rs3903239 likely affects the gene expression of *PRRX1*, resulting in atrial conduction, which prevents the development of AF, establishing it as a protective factor [21]. The prevalence of the rs2106261 variant (Table 2), located on chromosome 16q22 in the *ZFHX3* gene, is associated with an increased risk of AF. Among Asians, the rs2106261 SNP affects normal conductivity, making people more susceptible to atrial fibrillation (AF). Furthermore, rs2106261 is considered a key genetic marker for AF susceptibility, supported by research studies and meta-analysis [42,43]. The prevalence of rs7698692, a variant (Table 2) located near the *HAND2* gene on chromosome 4q34, is a susceptibility locus for AF, particularly among the Japanese or East Asian population [44,45]. Variants affecting the *HAND2* gene may alter the heart’s conduction system, thereby increasing the risk of AF. Though the prevalence of this variant is relatively low in the Indian population, its role in heart development and conduction system differentiation makes it a relevant risk factor for AF [45].

The prevalence of minor allele frequency was compared with the IndiGenome database. Comparative analysis reveals that the frequency of the minor allele T of the SNP rs2200733 increased from 13% (in the IndiGenome database) to 16% (in our study). Similarly, the frequency of the minor allele T at rs10033464 increased from 23% to 27%. Regarding rs13143308, the minor allele T decreased in frequency from 36% to 32%. However, in this study, for rs883079, the frequency of the minor allele T was reduced from 53% to 46%. For rs3903239, the frequency of the minor allele G increased slightly from 23% to 25%. As for rs2106261, the minor allele T maintains a constant frequency of 68% in both groups. For rs7698692, the frequency of the minor allele G moderately decreased from 16% to 14%. Thus, our findings suggest that specific SNPs showed increased frequencies, while others remained consistent (Table 5).

Furthermore, this study identified the varying prevalence of homozygous mutant genotypes for SNPs linked with AF in the young adult population in India (Table 3). The rs2200733 variant (*PITX2*) had a low prevalence, with 3% of individuals carrying the TT genotype. In contrast, the other *PITX2* gene variants, rs10033464 and rs13143308, had a high prevalence, with 10% carrying the TT genotype. Thus, the highest prevalence of these variants may suggest a strong correlation with AF susceptibility in young adults in the Indian population. The rs883079 (*TBX5*) variant is present in 21% of those carrying the TT genotype, suggesting genetic protection against AF. The rs3903239 (*PRRX1*) and rs2106261 (*ZFHX3*) variants, each with a frequency of 7% in the homozygous mutant GG and TT genotypes, respectively, are consistent with their roles in AF risk. The rs7698692 (*HAND2*) variant is rare, with only 1% of individuals having the GG genotype.

This study’s distribution of risk alleles reveals that a significant proportion of individuals (20%) possess four risk alleles. In comparison, high-risk allele groups comprise six (7%), seven (4%), or eight (3%) risk alleles. This implies that even young and healthy individuals carrying AF-related risk alleles emphasize the necessity of early genetic screening, particularly for those with a family history of AF or related cardiac conditions. Genetic counseling and preventive measures would be essential for these individuals to mitigate the onset of AF and its associated complications. As genetic predisposition plays a more prominent role in determining AF in younger adults [46], risk identification, increasing awareness, and conducting genetic testing are essential for early diagnosis and treatment. This study will contribute to implementing genetic screening for AF susceptibility in the future. This can be practically achieved by targeting the high-risk population with a family history of AF and early-onset AF using authenticated tools like polygenic risk scores or specific genetic variant panels.

Our study was conducted on a small group of young adults. Therefore, further studies are required on a larger cohort. Furthermore, to confirm the obtained observations, a larger population comprising diverse groups is essential to better understand the underlying factors contributing to allele frequency differences across various demographic and environmental contexts. The study has certain limitations, including a small sample size limited to a specific age range and locality within the Indian population, which does not represent the country adequately. A more representative cohort is needed, along with a study to enhance the generalizability of the results. A further limitation is that this study used a cross-sectional framework, which limits our ability to understand the causal associations between genetic variants and AF. In the future, case–control studies or longitudinal studies should be used to study the temporal relationships between and predictive properties of these SNPs. Since we studied individual SNPs rather than linkage disequilibrium (LD) haplotypes, we could not understand the genetic framework of AF. Functional studies are essential to evaluate the biological significance of these variants. As a result of incorporating diverse ethnic groups, we might be able to better understand these variants across a range of genetic backgrounds and enhance risk stratification models.

## 5. Conclusions

This study provided insight into the associations between genetic variants and atrial fibrillation (AF) in the young adult population in India. The significant distribution of a major proportion of risk alleles among young adults emphasizes the need for further research into the genetic predisposition of AF in this population. Understanding the associations between genetic factors and atrial fibrillation (AF) will enable early diagnosis and the development of personalized therapies, thereby mitigating the long-term burden of AF-related complications.

## Figures and Tables

**Table 1:** Single-nucleotide polymorphisms in AF-associated transcription factor genes with allelic variants, genomic location, and sequence context.

Gene	SNP ID	Allele Change	SNP Type	Context Sequence	TaqMan Assay Id
*PITX2*	rs2200733	C/T	Intergenic	GTGGTACTTGGGTTTTGATTTTGAT[C/T]AGAGAAAATTAGAACAGGTAATATT	C_16158671_10
*PITX2*	rs10033464	T/G	Intergenic	TTTTTACATTGTTAGAGTCAAGAAA[T/G]AAGTGCTTTCATCAAGCTCTGAGTT	C_30351088_30
*PITX2*	rs13143308	T/G	Regulatory region	GATGGATAAACCAAATGCATAAATA[G/T]CTTTGCCTTATTTCTAACCTCTTTG	C_31214706_10
*TBX5*	rs883079	C/T	3′UTR variant	ATTGGGTGAAATGAAAAATCTTGTC[C/T]GTGGGGTTCTCTTGGCTACTGTCTC	C_7526955_10
*PRRX1*	rs3903239	A/G	Intron	TTTCCTCTGCAATGTTTAATCTGCC[A/G]TTAATCCTGTCCATTGTATTTTTCA	C_8919547_10
*ZFHX3*	rs2106261	C/T	Intron	AGATAGAGCTCGTCCAGAGAATTGT[C/T]CAACCATCCATTAAAATATCCAAGT	C_16097559_10
*HAND2*	rs7698692	G/A	Intergenic	GGGACTTTTAATAAAACCACTTGGG[A/G]ATATTAATGTGATACGTAAGTGGCT	C_310074_10

**Table 2:** Demographic profile of the study population.

Demographic Characteristics	Young Adult Population, n = 250 (%)
Age (years) (mean ± SD)	25.81 ± 6.19
Male, n (%)	173 (69)
Female, n (%)	77 (31)
BMI (kg/m^2^) (mean ± SD)	24.9 ± 4.2
Normal, n (%)	125 (50)
Overweight, n (%)	96 (38)
Obese, n (%)	29 (12)
SBP (mmHg)	127.4 ± 15.3
DBP (mmHg)	84.8 ± 9.1
Alcohol consumption, n (%)	91(36)
Smoking, n (%)	52 (21)
Family History of Obesity, n (%)	42 (17)
Family History of Cardiovascular Disease, n (%)	38 (15)
Family History of Diabetics, n (%)	58 (23)

BMI: body mass index; SBP: systolic blood pressure; and DBP: diastolic blood pressure.

**Table 3:** Genotype and allele distribution of SNPs responsible for atrial fibrillation.

Gene	SNP ID	Genotype and Alleles	Study Population		95% CI	
			N = 250	Percentage (%)	Lower	Upper
*PITX2*	rs2200733	CC	177	71	60	82
		CT	65	26	20	33
		TT	8	3	1	6
		C	419	84	75	92
		T	81	16	12	20
	rs10033464	GG	138	55	46	65
		GT	88	35	28	43
		TT	24	10	6	14
		G	364	73	65	80
		T	136	27	22	32
	rs13143308	GG	117	47	38	56
		TG	107	43	35	51
		TT	26	10	6	15
		G	341	68	61	75
		T	159	32	27	37
*TBX5*	rs883079	CC	71	28	22	35
		CT	127	51	42	60
		TT	52	21	15	27
		C	269	54	47	60
		T	231	46	40	52
*PRRX1*	rs3903239	AA	144	58	48	67
		AG	88	35	28	43
		GG	18	7	4	11
		A	376	75	67	83
		G	124	25	20	29
*ZFHX3*	rs2106261	CC	138	55	46	65
		CT	94	38	30	46
		TT	18	7	4	11
		C	370	74	66	81
		T	130	26	21	30
*HAND2*	rs7698692	AA	184	74	63	85
		AG	63	25	19	32
		GG	3	1	0	3
		A	431	86	78	94
		G	69	14	10	17

CI: confidence interval; data were represented as numbers (%).

**Table 4:** Comparison of the variant allele frequency of AF-associated variants in South Asian population with databases.

Chr:Position:	rsID	Gene	Variant Allele	1000 Genome	gnomAD	IndiGenome	In Our Study
chr4-110789013	rs2200733	*PITX2*	T	0.1309	0.1338	0.1320	0.16
chr4-4110799605	rs10033464	*PITX2*	G	0.8006	0.7767	0.7706	0.74
chr4-110793263	rs13143308	*PITX2*	G	0.6759	0.6527	0.6466	0.68
chr12-114355435	rs883079	*TBX5*	T	0.5133	0.5198	0.5034	0.46
chr1-170600176	rs3903239	*PRRX1*	G	0.2168	0.2232	0.2373	0.25
chr16-73017721	rs2106261	*ZFHX3*	T	0.2393	0.2298	0.2627	0.26
chr4-173682953	rs7698692	*HAND2*	A	0.8793	0.8775	0.8483	0.86

**Table 5:** Distribution of risk alleles in the study population with a 95% confidence interval.

Number of Risk Alleles	Study Population		95% CI	
	N = 250	Percentage (%)	Lower	Upper
0	7	3	1	5
1	23	9	5	13
2	37	15	10	20
3	48	19	14	25
4	50	20	14	26
5	48	19	14	25
6	18	7	4	11
7	11	4	2	7
8	8	3	1	6

CI: confidence interval; data were represented as numbers (%).

## Data Availability

All data will be available from the corresponding author upon reasonable request.

## Supplementary Materials

The following supporting information can be downloaded at: https://www.mdpi.com/article/10.3390/medicina61050900/s1. Detailed information on the genotyping results with the HWE values of the study group is shown in the Supplementary Table S1.

## Abbreviations

AF	Atrial fibrillation
SNP	Single-nucleotide polymorphism
BMI	Body mass index
PCR	Polymerase Chain Reaction
PITX2	Paired-Like Homeodomain 2
TBX5	T-Box Transcription Factor 5
PRRX1	Paired Related Homeobox 1
ZFHX3	Zinc Finger Homeobox 3
HAND2	Heart and Neural Crest Derivatives Expressed 2
OR	Odds ratio
CI	Confidence interval
SPSS	Statistical Package for the Social Sciences
