# Supplementary material for: Prevalence of Genetic Variants Associated with Atrial Fibrillation Risk in the Asymptomatic Young Adult Population
Source: Medicina (Kaunas). 2025 May 15;61(5):900. doi: 10.3390/medicina61050900 (PMC12112815; doi:10.3390/medicina61050900)
Supplement: Supplementary file 1 [file medicina-61-00900-s001.zip › medicina-3594605-supplementary.pdf]

Supplementary Table S1. Hardy–Weinberg equilibrium of selected SNP genotype distribution frequency in the control group.

Variant ID	Major/Minor	Expected			Observed			X2	p-Value
rs2200733	C/T	CC	CT	TT	CC	CT	TT	0.449	0.502
		175.6	67.9	6.6	177	65	8		
rs10033464	G/T	GG	GT	TT	GG	GT	TT	3.09	0.078
		132.5	99.0	18.5	138	88	24		
rs13143308	G/T	GG	GT	TT	GG	GT	TT	0.043	0.833
		116.3	108.4	25.3	117	107	26		
rs883079	C/T	CC	CT	TT	CC	CT	TT	0.119	0.729
		72.4	124.3	53.4	71	127	52		
rs3903239	A/G	AA	AG	GG	AA	AG	GG	0.791	0.373
		141.4	93.2	15.4	144	88	18		
rs2106261	C/T	CC	CT	TT	CC	CT	TT	0.130	0.717
		136.9	96.2	16.9	138	94	18		
rs7698692	A/G	AA	AG	GG	AA	AG	GG	0.876	0.349
		185.8	59.5	4.8	184	63	3		
